# Developing and piloting an interactive syphilis surveillance dashboard through user-centred design: An implementation report from Interior Health, British Columbia

**DOI:** 10.14745/ccdr.v52i78a06

**Published:** 2026-07-30

**Authors:** Temuulen Enebish, Barbara Gauthier, Jonathan Malo

**Affiliations:** 1 Canadian Field Epidemiology Program, Centre for Emergency Preparedness, Public Health Agency of Canada, Ottawa, ON; 2 Interior Health, Kelowna, BC

**Keywords:** syphilis, surveillance, dashboard, user-centred design, implementation science, public health informatics

## Abstract

**Background:**

Syphilis rates have risen in Canada and British Columbia (BC), increasing the need for timely, small-area intelligence. In BC, 1,983 cases of infectious syphilis were reported in 2024, representing a rate of 34.8 cases per 100,000 population. An additional 27 cases of congenital syphilis were also confirmed. Routine quarterly reporting limits responsiveness when incidence shifts quickly.

**Objective:**

To describe the development, implementation, and early evaluation of an interactive surveillance dashboard for syphilis in Interior Health (IH), delivering monthly indicators at the Local Health Area (LHA) level.

**Methods:**

An internal dashboard was developed using user-centred design and iterative stakeholder engagement. The dashboard integrates case, testing, and denominator data to display monthly indicators, 12-month trends, and LHA-level patterns, with role-based access and privacy safeguards for authorized users.

**Results:**

Perceived usefulness, simplicity, and acceptability were assessed using Likert ratings and structured feedback. In a pilot with 11 users across key IH program groups, overall impression, ease of use, usefulness, and program fit were rated 5.0/5, 4.9/5, 4.9/5, and 4.6/5 (n=8–9). Users described value for prioritizing LHAs, planning interventions, and briefing decision-makers.

**Conclusion:**

An interactive, LHA-level dashboard was feasible within existing systems and acceptable to pilot users, with perceived improvements in timeliness and clarity. Effects on downstream public health outcomes remain to be evaluated. The approach may be transferable to similar jurisdictions.

## Introduction

Syphilis incidence has risen sharply in Canada and British Columbia (BC) over the past decade (1,2). Provincial rates remained below three per 100,000 throughout the early 2000s before beginning a sustained increase. Infectious syphilis cases reached 1,428 in 2021 (27.5 per 100,000), the highest in 40 years (2), and increased further to 1,983 in 2024 (34.8 per 100,000) (3). Congenital syphilis underscores the public health importance of rising transmission among females of reproductive age. In BC, female infectious syphilis cases have been strongly shaped by concurrent housing instability, substance use, and mental illness, and maternal cases often involve recent sexually transmitted infection (STI) history and factors that may affect access to prenatal care (4). The 27 cases confirmed in BC in 2024 highlight the continuing importance of prevention, timely testing, and early antenatal diagnosis (3). Within Interior Health (IH), one of BC’s five regional health authorities serving approximately 830,000 residents in the southern interior, cases increased from 54 in 2019 to 228 in 2024, representing a 278% increase in the rate over the same period (5).

Timely, small-area intelligence is essential for regional authorities to monitor transmission, identify emerging clusters, and allocate resources, yet routine quarterly and annual static products leave gaps in responsiveness. In IH, review previously relied on provincial quarterly reports supplemented by ad hoc analyst-generated tables to obtain Local Health Area (LHA) detail, limiting timely interpretation and requiring repeated data preparation. Local Health Areas are BC’s standard sub-regional geography for service planning, typically containing 5,000–60,000 residents. Digital public health dashboards can support timely situational awareness, but their usefulness depends on clear indicator definitions, regular refresh cycles, and usability and governance considerations (6). Pairing testing volumes and positivity with case counts further supports targeted action beyond passive case reporting. In BC, centralized testing through the BC Centre for Disease Control (BCCDC) Public Health Laboratory provides complete testing data, an enabler not available in all jurisdictions.

Peer-reviewed descriptions of dashboard development, governance, and early-stage evaluation in Canadian regional public-health contexts remain rare. Publishing this early evaluation lets other jurisdictions learn from replicable design and governance choices before long-run effectiveness data are available. The objectives of this early evaluation were to: 1) describe the user-centred design (UCD) and governance approach used to develop the dashboard; 2) describe its implementation within IH’s existing surveillance infrastructure; and 3) assess perceived usefulness, simplicity, and acceptability among primary users after the initial pilot period.

## Methods

### Setting and participants

The dashboard was implemented as an internal decision-support tool for IH teams involved in syphilis prevention and response: Epidemiology and Surveillance, Communicable Disease, Sexual and Reproductive Health, Medical Health Officers, and senior public health decision-makers. Outputs are provided for IH residents with infectious syphilis at the LHA level, with privacy safeguards applied throughout. Eleven primary users were purposively sampled and invited by email to participate voluntarily. Nine completed the Likert ratings, and two additional participants provided qualitative feedback without completing the questionnaire.

### Dashboard development

Development followed a UCD process with end users across the program groups described above. In the discovery phase, semi-structured interviews and workflow mapping identified core use cases (e.g., routine monitoring, situational awareness, planning) and pain points (e.g., fragmented information sources, static reports, inconsistent refresh cycles). In the subsequent iterative phase, low-fidelity mock-ups, including simple wireframes and greyscale click-throughs used to test information architecture before coding, were reviewed collaboratively in short cycles. Brief usability assessments of evolving prototypes were conducted, and feedback was incorporated into successive releases. The process converged on three core view types: 1) an overview page anchored by a concise key performance indicator (KPI) tile row; 2) dedicated trend views; and 3) small-area LHA analysis views.

Design choices emphasized clear labels, predictable filters, and responsive performance (page load and filter responses under approximately three seconds on typical analyst laptops over the corporate network).

### Implementation

The dashboard was built as an R Quarto dashboard rendered to Hypertext Markup Language (HTML), and published in a secure internal IH environment with monthly data refreshes. It integrates three data streams: the infectious syphilis case line list (stage, key event dates, geography), monthly laboratory testing volumes by LHA, age, and sex, and population denominators. Postal codes from the case line list are mapped to LHAs using the BC Stats standardized conversion file ensuring that case, testing, and denominator data align to the same boundaries and that rates and positivity are computed consistently across streams. Because of data access restrictions for sensitive STI data, surveillance data are manually extracted and curated each month with quality checks applied during refresh. The refresh currently requires approximately two hours of dedicated analyst time per month for extraction, quality assurance, and publication, performed by one epidemiologist with backup coverage.

**Figure 1:**
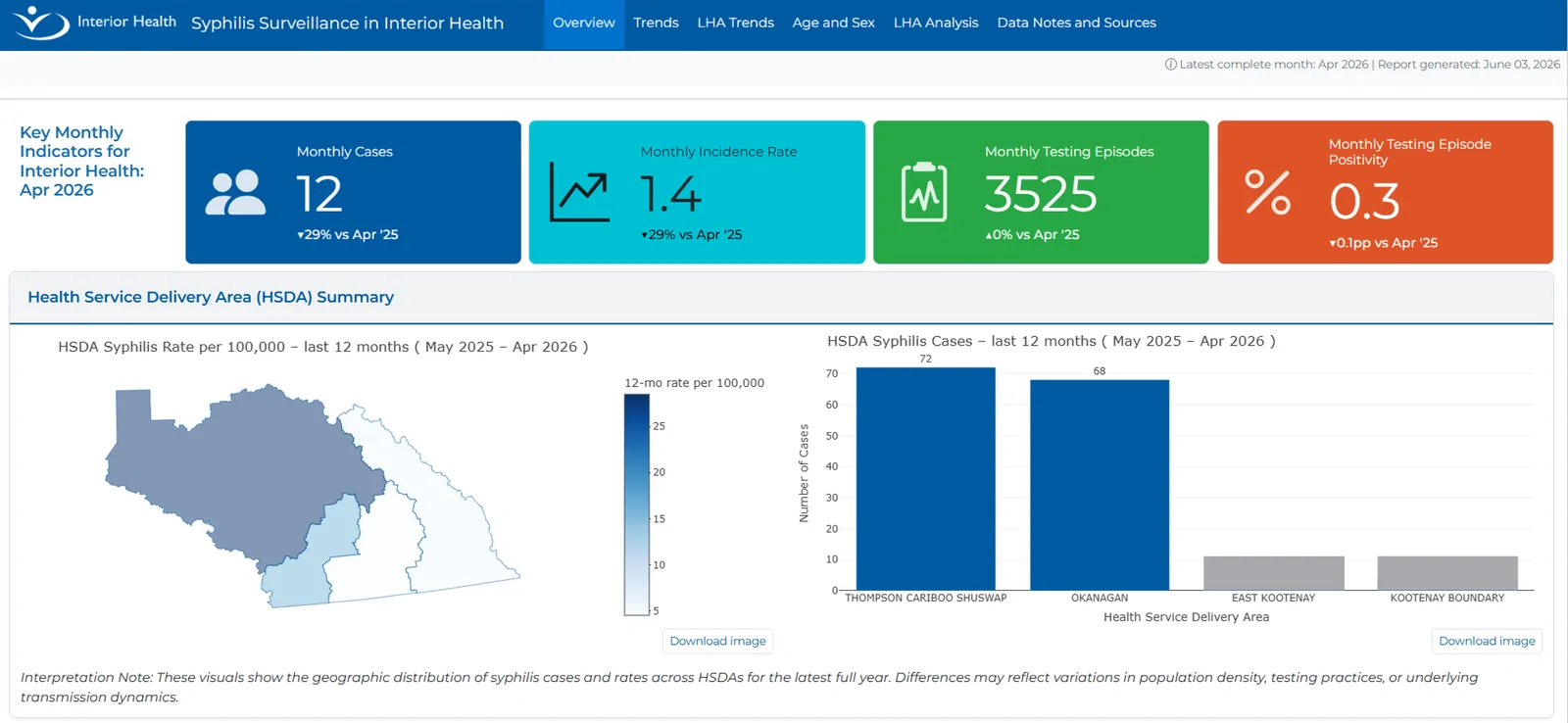
Dashboard overview Note: This figure shows an internal dashboard view and is presented for illustration only

The dashboard includes an Overview page with KPI tiles for monthly cases, incidence rate, testing episodes, and testing episode positivity plus Health Service Delivery Area (HSDA) summary visuals (**Figure 1**), alongside separate trends, LHA Trends, Age and Sex, and LHA Analysis pages. Export functions for filtered aggregates and figures support briefing work; indicator definitions and the refresh standard operating procedure are in supplementary materials (**Table 1** summarizes capabilities).

**Table 1:** Data sources and dashboard capabilities

Data stream	Source	Key fields	Primary dashboard use
Infectious syphilis cases	Provincial case line list (IH residents)	Stage, dates, postal code, sex, age	Official surveillance counts, incidence rates, trends
Laboratory testing	Public Health Laboratory (BCCDC)	Test counts by month, LHA, age, sex	Testing episodes, testing episode positivity
Population estimates	BC Stats	Population by LHA, age, sex	Rate denominators

Abbreviations: BC, British Columbia; BCCDC, British Columbia Centre for Disease Control; IH, Interior Health; LHA, Local Health Area

### Governance framework

Governance measures prioritized privacy while maximizing operational utility. Access to the dashboard is role-based, with user agreements specifying permitted uses. Because the dashboard is restricted to authorized internal users, counts are displayed without routine small-cell suppression. However, LHA rates based on fewer than 20 cases are flagged as unstable with guidance on cautious interpretation. Maps aggregate to LHA to minimize re-identification risk. Data are drawn from authoritative internal sources within existing privacy frameworks, including the BC *Freedom of Information and Protection of Privacy Act* (FOIPPA) (7). An advisory group of epidemiology, privacy, and program representatives reviewed indicator definitions, refresh cadence, rate-stability guidance, and release practices, providing lightweight change control. Accessibility followed web content accessibility guidelines (WCAG) guidance (8).

### Outcome measures

Addressing objective 3, an early pilot evaluation was conducted with the 11 primary users aligned with standard surveillance evaluation attributes (9,10). Attributes most relevant to early implementation and routine use (usefulness, simplicity, acceptability) were prioritized. A structured feedback guide was used to collect Likert ratings on overall impression, ease of use, usefulness, and program fit, with prompts on examples of use, most valuable feature, and requested changes. Items were rated on a 5-point scale (1=strongly disagree or very poor; 5=strongly agree or excellent). Feedback was requested on September 9, 2025, and responses were received over the following month, after users had access to the dashboard for a median of approximately four weeks. Nine participants completed the Likert ratings, and two additional participants provided qualitative feedback only. Both forms of feedback were incorporated into the thematic synthesis. An informal visual review of layout, labelling, and readability was also conducted, and processed data files were verified to contain no personal identifiers. However, a formal expert usability evaluation was not conducted. Ratings were summarized descriptively and open-ended responses were synthesized into themes. Formal pre-post comparison of timeliness and decision-making efficiency against prior static products was out of scope for the early pilot because baseline metrics were not captured pre-implementation. Capturing these metrics is planned for the three- to six-month follow-up, which will quantify sustained adoption and timeliness (e.g., days from month-end to publication).

### Ethics review

This work consisted of routine public health surveillance plus an early service-evaluation component. The dashboard itself is a routine surveillance product conducted under the *Public Health Act* within IH’s data stewardship framework and does not display personal identifiers. The component relevant to project ethics screening was the informal feedback collection from project team members, which under IH practice would fall under the A pRoject Ethics Community Consensus Initiative (ARECCI) project-level screening rather than full research ethics board review. Participation was voluntary, feedback is reported in aggregate, and quotes are de-identified.

## Results

### Primary outcomes

Addressing objective 3, nine of the eleven invited primary users completed the Likert ratings across the five stakeholder groups, and two additional users contributed qualitative feedback. Ratings were strong (**Table 2**): overall impression 5.0/5 (n=9), ease of use 4.9/5 (n=9), usefulness 4.9/5 (n=9), and program fit 4.6/5 (n=8).

**Table 2:** Pilot ratings summary (Likert scale 1.0–5.0)

Attribute	Mean (1.0–5.0)	n
Overall impression	5.0	9
Ease of use	4.9	9
Usefulness	4.9	9
Program fit	4.6	8

### Secondary outcomes

Structured feedback highlighted the dashboard’s clarity, ease of navigation, and value for consolidating information previously scattered across multiple sources. Users reported that filterable trends and geographic views improved routine situational awareness and focused discussion during monitoring meetings. One clinician wrote: “Seeing the areas that are most affected… and the trends for the areas where we have implemented more testing options and more enhanced follow up… are our interventions working or not?” Suggestions focused on minor usability refinements (e.g., an abbreviations box, sticky table headers, higher-contrast bars, improved layout on smaller screens).

Pilot users also perceived improved timeliness of routine monitoring and clearer communication of recent changes to leadership during the pilot period. Local Health Area views supported prioritization for outreach and partner services, and monthly trends helped participants monitor patterns relevant to intervention planning between provincial quarterly cycles. One participant noted: “If we are seeing more cases in a particular area… I can see if the positivity or cases are actually increasing to help advocate for additional services.” One-click image export reduced ad hoc preparation for recurring planning artifacts. These perceived improvements will be quantified against baseline workflow measures at follow-up.

### Intervention experience

Three practical challenges emerged during iterative development and were corroborated in pilot feedback: small-area volatility required careful visual cues to avoid over-interpretation (addressed with consistent denominators, rolling windows, and guidance notes); harmonizing data definitions across streams needed upfront effort and a governance cadence; and balancing accessibility with privacy required iterative parameter setting with stakeholder input. None of these impeded early use, but they informed the roadmap and evaluation design.

## Discussion

### Key findings

Addressing objectives 1 and 2, an interactive syphilis surveillance dashboard was developed and implemented integrating monthly indicators, 12-month trends, and LHA-level spatial patterns. The UCD process and governance approach enabled rapid iteration while maintaining privacy safeguards and accessibility. Addressing objective 3, early use suggests the tool is acceptable and useful for routine monitoring and planning, with pilot users reporting perceived improvements in timeliness and clarity of information flow.

### Comparisons

The approach that was used aligns with published guidance on rapid-cycle, user-centred methods for population health surveillance dashboards, which emphasize iterative prototyping with end users, clear indicator definitions, and lightweight governance over lengthy upfront specification (6,11). Expanding provincial quarterly products with LHA-level views would likely have addressed some information needs, but it would have been less able to address the interactivity and refresh-cadence gaps stakeholders identified and would still have required analysts to assemble ad hoc briefing materials. The dashboard is designed for internal operational use with role-based access and privacy safeguards, distinguishing it from publicly available STI dashboards, such as Centers for Disease Control and Prevention AtlasPlus, (12) and making it suited to small-area program management between provincial reporting cycles.

### Implications for practice

For regional public health teams, a lightweight, interactive dashboard may streamline routine monitoring, shift effort from data preparation to interpretation, and strengthen cross-program communication. Early engagement and frequent feedback cycles were critical to aligning features with actual workflows. The approach used may be adaptable to other communicable diseases (e.g., gonorrhea, chlamydia) and jurisdictions with similar data streams, governance structures, and analytic capacity. Potentially transferable elements include the UCD workflow, the Overview page layout shown in Figure 1, the use of separate Trends, LHA Trends, Age and Sex, and LHA Analysis pages, and the privacy and access governance pattern. Integrating testing volumes and testing episode positivity with case counts can support targeting of areas with low testing uptake, moving beyond passive case surveillance toward more actionable intelligence for intervention planning.

### Limitations

Limitations align with the evaluation objectives. Regarding objectives 1 and 2, the dashboard reflects a single regional context and may require adaptation elsewhere. Manual monthly data extraction, required because of privacy and access restrictions for STI surveillance data, limits refresh frequency relative to automated pipelines and is resource-intensive to sustain. Jurisdictions considering similar approaches should weigh available analytic capacity against competing public health priorities. Secure automated data integration within existing privacy frameworks is planned for future work.

Regarding objective 3, findings are based on self-reported perceptions from a small sample of early adopters who were purposively invited and already engaged with surveillance modernization. This may have biased ratings upward and limited generalization to less-engaged users. The evaluation measured perceptions rather than behavioural change or downstream outcomes, and was conducted after a short period of use, so quantitative adoption and satisfaction metrics are preliminary. Attributes like timeliness benchmarks and data-quality dimensions require longer observation windows. Because baseline workflow measures were not captured pre-implementation, reported improvements in timeliness and decision-making are perceived rather than demonstrated. Effects on downstream outcomes, such as case finding and congenital syphilis prevention, were not formally evaluated and are planned for follow up in three to six months.

### Data availability

The underlying case and laboratory datasets contain personal health information and are not publicly shareable. Requests for aggregated indicators and metadata may be considered by IH, subject to applicable governance and privacy review (7).

## Conclusion

A UCD process and iterative engagement of end users produced an interactive, LHA-level syphilis surveillance dashboard tailored to routine surveillance needs and decision-making, while prioritizing privacy and accessibility. Early pilot feedback indicated perceived improvements in timeliness and clarity of information flow, suggesting that UCD can be a useful approach to modernizing public health surveillance at a regional scale. Demonstrated effects on downstream public health outcomes remain to be evaluated. The approach may be adaptable to other conditions and jurisdictions with similar data streams, governance structures, and analytic capacity.
